# Comparison of approaches for quantifying extractables in a variety of polymeric medical device materials with LC-MS/MS

**DOI:** 10.1007/s00216-026-06471-9

**Published:** 2026-03-31

**Authors:** Anneke L. Niehuus, Max Renneisen, Sascha Reinschmiedt, Denise Sievers, Oliver J. Schmitz, Sven W. Meckelmann

**Affiliations:** 1 Drägerwerk AG & Co. KGaA, Moislinger Allee 53-55, 23558 Lübeck, Germany; 2 Applied Analytical Chemistry, University of Duisburg-Essen, Universitätsstrasse 5, 45141 Essen, Germany

**Keywords:** Quantification, Extractables, Polymer additives, Response factor, Matrix effects, Polymer matrix

## Abstract

Quantification by LC-MS using electrospray ionization (ESI) is challenging due to various factors that influence signal intensity. The gold standard involves the use of isotopically labelled standards spiked into the sample. However, for the analysis of extractable compounds from polymeric materials in medical devices, such standards are often unavailable. Nevertheless, these extractables must be monitored and quantified in accordance with regulatory requirements. In this study, we compared different LC-MS-based approaches for quantifying extractables from polymers commonly used in medical devices. The evaluation focused on 25 analytes, partially of high regulatory relevance under the European Medical Device Regulation. Two primary strategies were investigated: quantification using internal isotope-labelled surrogates, and the use of relative response factors (RRFs) from an experimentally established database. Using the closest eluting internal surrogate resulted in a mean quantification error of over threefold, which improved to 2.7-fold when surrogates were selected based on structural similarity for ESI+ mode while no significant improvement was observed for ESI- mode. In contrast, employing the RRF database yielded a lower bias, with a mean error of 1.6-fold (ESI+) and 2.3-fold (ESI-) using structurally similar compounds and as low as 1.2-fold when the analyte’s specific RRF was available. These approaches were applied to 22 polymer materials spanning 8 polymer types. Each was extracted using three different solvents and the impact of matrix effects on quantification accuracy was assessed. Although matrix effects such as ion suppression were observed, their influence was minor compared to the differences among the quantification approaches.

**Graphical Abstract:**

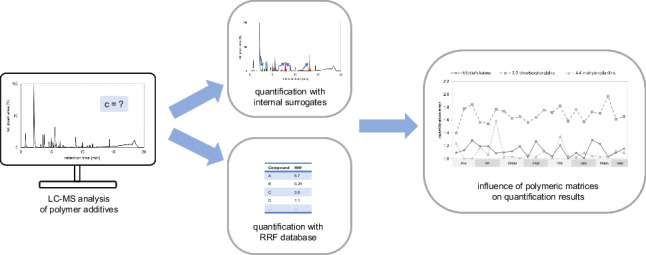

**Supplementary Information:**

The online version contains supplementary material available at 10.1007/s00216-026-06471-9.

## Introduction

Polymers are key materials for the construction of various medical devices. They have the advantage of being cheap, lightweight, and highly versatile [1], and are a broad group of different materials encompassing (among others) thermoplastics, elastomers, and thermoplastic elastomers. Thermoplastics melt at higher temperatures, allowing for easy molding, and harden on cooling. To obtain the desired qualities, additives such as antioxidants, plasticizers, lubricants or flame retardants are added. In contrast, elastomers are typically soft, flexible, and can recover completely from high extensions. The amount of additive in an elastomer can be as high as 50% or more of the total mass since they can contain many fillers needed for reinforcement, as well as vulcanizing agents for cross-linking and other additives. As their name states, thermoplastic elastomers are a hybrid between the former two classifications. They are as flexible as elastomers but can be easily processed and molded via heating like thermoplastics [2–4]. Regardless of the type of polymer, the used additives are mostly not chemically bound to the polymer and may leach out, along with other components such as residual monomers, reaction products or impurities. These compounds are relevant from a toxicological point of view as they are in close contact to patients when used in medical applications. Therefore, it is essential to know the composition of these materials for further evaluation and risk assessment, especially when used in medical devices.

For the assessment, two classes of compounds can be considered, the so-called extractables and leachables (E&L). Extractables are compounds that can be extracted from polymers by suitable solvents, often using harsher than realistic conditions such as higher temperatures and longer extraction times. Leachables are considered when evaluating the biocompatibility. Here, it is assessed which compounds might leach out during everyday application of the medical device [5]. Often, it is necessary to go a step further and evaluate the entire polymer regardless of what will realistically leach out. This is especially relevant for environmental issues and questions of sustainability, but it is also part of numerous regulations worldwide, such as the European Medical Device Regulation (MDR). The MDR restricts the presence of more than 1000 substances above a threshold of 0.1% (w/w) in polymeric material [6]. Since the composition of polymers is often confidential, and the impurities and reaction products might not necessarily be known by the manufacturers, sophisticated instrumental analysis is imperative to manufacture safe medical devices.

Chromatography coupled to mass spectrometry (MS) is the method of choice for analyzing polymer extracts. Chromatography enables the separation of complex polymer matrix, and identification can be achieved by mass spectrometry, either by comparing the spectra to libraries or, in the case of high-resolution (HR)-MS, by determining the accurate mass and calculating possible chemical formulas. Currently, there is no gold standard, and both liquid chromatography (LC) as well as gas chromatography (GC) are applied to characterize organic polymer additives. A well-established method for E&L characterization is pyrolysis-GC-MS which requires little to no sample preparation. The polymer is heated to high temperatures, and the thermal degradation products are analyzed. While this method is simple and reliable, it is mainly useful to get qualitative rather than quantitative information on the polymer composition [7]. Complementary to these GC methods, LC analysis is needed to further evaluate semi-volatile (SVOCs) and non-volatile organic compounds (NVOCs). Coupled to HRMS, the required sensitivity is reached, and the high mass resolution allows for accurate mass determination which is needed for non-target analysis of extractables [8–11].

Besides the identification of the various compounds, a quantitative assessment is necessary for toxicological risk assessment. A direct quantification is often not feasible because of the high amount of emerging extractables in the samples. Another important aspect is that authentic reference standards are often not available for purchase. This is even more critical when considering that many of the substances are restricted by the MDR or other regulations. However, accurate quantification is not required, and less sophisticated approaches are usually sufficient. An assessment allows to provide information on whether or not identified extractables are above the threshold of 0.1% (w/w). Quantification of extractables without authentic reference standards is not trivial. Electrospray ionization (ESI) sources are frequently used in LC-MS due to their versatile application. However, it is well described that there can be a huge variation in response, even for structurally similar analytes [12–14]. Furthermore, the mechanism of ESI is easily influenced by matrix effects, leading to either ion suppression or ion enhancement [15–17] and making accurate quantification extremely difficult.

In recent years, various approaches for quantifying without authentic reference standards have been proposed and tested, often incorrectly referred to as “semiquantification”. Typically, analytes are quantified by so-called surrogates which can be added to the sample and are often isotope-labelled. The surrogate that will be used for quantification can be chosen according to similar retention time (RT) or similar structure since the response with an ESI source is structurally specific and can change with the RT [18]. The advantage of this approach is its simplicity [19], especially when quantifying with the closest eluting surrogate since the analyte does not need to be identified priorly. While quantification errors can be as high as 10 times, the developed quantification approaches usually yield satisfying results for the intended application. Pieke et al. [20] reached mean errors as factors of 2.1 (ESI+) and 1.7 times (ESI-) when quantifying compounds in food contact material with close-eluting surrogates, while all of the analytes could be quantified with an error below 4.0 times. Evans et al. [18] reached mean errors of 3.1 times with this approach, and 70% of the analytes could be quantified with an error below 2.0 times. When compared, it could be shown that using a structurally similar surrogate yielded slightly better results for the chosen set of analytes [21].

Another approach is to establish a database containing the response factors (RF) of analytes in relation to the RF of an internal standard (relative response factor (RRF)). The RF is calculated by dividing the peak area by concentration (see formulas S1 and S2 in the supplementary material). The known RRF can therefore be used to rapidly quantify the analyte in a sample. This approach has been mainly reported for GC-MS [22, 23] but it has also been applied to a LC-MS method [24]. It is less time-consuming and more cost-efficient than directly quantifying each analyte via calibration curves. A major drawback, however, is the variation of the RRFs over time and between laboratories, so that remeasurements in defined intervals are necessary. Another disadvantage is that authentic reference standards are needed. A solution could be to determine the RRFs of a “marker set” of standards, and to match the analytes to those markers (external surrogates) according to their properties such as structural similarity. This allows use of a wider range of surrogates compared to adding (isotope-labelled) surrogates internally to the samples.

To objectively measure the structural similarity between two compounds for the quantification, 2D-based descriptors such as the sequence of atom pairs (AP) or the maximum common substructure (MCS) can be used. These descriptors are then used to calculate the similarity with the Tanimoto coefficient within a range of 0 to 1, where a higher Tanimoto score means a higher similarity [25]. These similarity calculations are typically used when environmental transformation products are supposed to be quantified via the parent compounds [26].

The focus of this work is the development of a concept for the quantification of a variety of extractables in different polymer matrices used in medical devices, with the goal of obtaining comprehensive information on the composition of these polymeric materials. The resulting quantification errors of different approaches are compared, and the effect of 22 different polymers extracted with three different solvents is tested. It is evaluated whether there are quantification approaches which are more subject to effects arising from polymeric matrices. The concept is further tested by quantifying analytes in a certified E&L screening mix.

## Experimental

### Chemicals and reagents

A set of 25 standards was obtained in the highest available purity. 1-Naphthol (CAS-No. 90-15-3), 2,2′-methylenebis(4-methyl-6-*tert*-butylphenol) (119-47-1), 2,4-tolylenediamine (95-80-7), 2-isopropylphenol (88-69-7), 2-naphthylamine (91-59-8), 2-phenylphenol (90-43-7), 2-*tert*-butylphenol (88-18-6), 3-chlorophenol (108-43-0), 3-nitrophenol (554-84-7), 4,4′-methylenebis(2-chloroaniline) (101-14-4), 4,4′-methylenebis(2-methylaniline) (838-88-0), 4,4′-oxydianiline (101-80-4), 4-isopropylbenzoic acid (536-66-3), 4-*tert*-butylbenzoic acid (98-73-7), Aniline Yellow (60-09-3), Basic Violet 3 (548-62-9), bisphenol A (80-05-7), Disperse Blue 1 (2475-45-8), Irgacure 907 (71868-10-5), and Michler’s ketone (90–94-8) were obtained from Merck KGaA (Darmstadt, Germany). 2,4,6-Tri-*tert*-butylphenol (732-26-3), 3,3′-dimethoxybenzidine (119-90-4), 3,3′-dimethylbenzidine (119-93-7), 4,4′-methylenedianiline (101-77-9), and 4-aminobiphenyl (92-67-1) were purchased from LGC Ltd (Teddington, UK). While many more compounds are restricted by the MDR, these analytes were chosen because of their plausibility to be found in polymers, their availability for purchase, and their analyzability by LC-MS. Structural information can be found in the supplementary material (Table S1).

Isotope-labelled standards were chosen as internally added surrogates. 1,3-Diphenylurea-D_10_ and *m*-toluidine-D_3_ were both purchased from LGC Ltd. (Teddington, UK), whereas diphenylamine-D_10_ was purchased from Cambridge Isotope Laboratories, Inc. (Tewksbury, MA, USA) and dicyclohexylphthalate-D_4_ as well as stearic acid-D_35_ from Merck KGaA (Darmstadt, Germany). 2,6-Dimethylphenol-D_3_, 2-chlorophenol-D_4_, 2-nitrophenol-D_4_, butylhydroxytoluene-D_21_, and phenoxybenzoic acid-^13^C_6_ were obtained from HPC Standards GmbH (Cunnersdorf, Germany). The choice was made due to the variety in structure, retention time, and response factor.

Formic acid and all solvents, including 2-propanol, acetone, acetonitrile, and toluene, were purchased from VWR International (Radnor, PA, USA) in LC-MS grade. Ammonium hydrogencarbonate was obtained from Merck KGaA (Darmstadt, Germany). Ultrapure water was desalted and bidistilled by a bidistillation apparatus (Quarzglas Komponenten und Service QCS GmbH, Maintal, Germany).

### LC-QTOF-MS/MS analysis

Liquid chromatography was performed with an Agilent 1290 Infinity II series coupled to an Agilent 6546 quadrupole-time of flight (Q-TOF) mass spectrometer (Agilent Technologies, Inc., Santa Clara, CA, USA). For the analysis in positive ion mode (ESI+), 0.5 µL of sample was injected onto an Agilent Eclipse Plus C18 column (2.1 × 100 mm; 1.8 µm). The column oven temperature was set at 40 °C. Water with 0.1% formic acid (A) and acetonitrile (B) were used as mobile phases in a gradient elution with a flow rate of 0.3 mL/min. The gradient started at 5% B held for 1 min, then changed linearly to 100% B in 18 min and held for 2 min. For reequilibration, the initial composition was set for 5 min. Total run time was 25 min. In negative ion mode (ESI-), 5 µL was injected onto an Agilent Extend C18 column (2.1 × 100 mm; 1.8 µm). Water with 8 mM ammonium hydrogen carbonate was used as eluent A instead while the rest of the method stayed the same.

The Dual Agilent Jet Stream source was used in ESI+ mode with the following settings: gas temperature at 320 °C, gas flow at 8 L/min, nebulizer pressure at 35 psi, sheath gas temperature at 350 °C, sheath gas flow at 11 L/min, capillary voltage at 3500 V, and nozzle voltage at 1000 V. For ESI- mode, the gas temperature was changed to 125 °C and the capillary voltage to 1500 V. Experiments were run in Auto-MS/MS mode in a range of m/z 50 to 1700 and with a scan rate of 10 spectra/s. Collision energies of 10 V, 20 V, and 40 V were set for the fragmentation of the precursors. Per cycle, a maximum number of 10 precursors were chosen, with an absolute threshold of 2000 counts and a relative threshold of 0.01%. An active exclusion was added after the recording of 2 spectra, and released after 0.5 min. A target value of 25,000 counts per spectrum was set for the abundance dependent accumulation.

### Sample preparation

In total, 22 samples from 8 polymer types were extracted. Chosen as representatives for thermoplastics were three polyamides 6 (PA6), three polypropylenes (PP), two polymethyl methacrylates (PMMA), and three acrylonitrile butadiene styrenes (ABS). Representing the class of elastomers were three ethylene propylene diene monomer (EPDM) rubbers, three fluoroelastomers (FKM) as well as two silicone rubbers (VMQ). In addition, three different thermoplastic elastomers (TPE) were selected. The exact composition of these polymeric materials was unknown, but they all find use in real medical devices.

The polymers were milled with an IKA A11 basic analytical mill (IKA Werke GmbH & Co. KG, Staufen im Breisgau, Germany). Around 500 mg of the particles were weighed into extraction thimblets and extracted via a Soxhlet extraction using the Universal Extractor E-800 from Büchi Labortechnik AG (Flawil, Switzerland) with 100 mL solvent for 100 cycles. The extraction conditions were optimized prior to this study to ensure exhaustive extraction according to ISO 10993–18. Water was used as a polar solvent, 2-propanol as a semi-polar, and toluene as a non-polar solvent. At the end of the extraction, the majority of the solvent was automatically evaporated and the concentrated extract was filled up to 50 mL with the respective solvent (apart from the toluene extracts which were filled up with 2-propanol instead). If the extract was cloudy or particles were visible, it was centrifuged, and the supernatant was transferred to a new vial. For each polymer sample, a double extraction was conducted. This procedure resulted in a total amount of 66 different extracts.

Stock solutions of the analytes were prepared in 2-propanol or acetone, depending on the solubility, in concentrations of 100 µg/mL. Each of the extracts was spiked with the analyte mix at a final concentration of 300 ng/mL in the extract and isotope-labelled standards (200 ng/mL). The pure solvents were also spiked for comparison.

To validate the quantification concept, the “Extractables and Leachables Screening Standard for LC” (Merck KGaA, Darmstadt, Germany) was measured and quantified as a certified reference material. It contained 21 analytes in acetonitrile: 1,3-di-*tert*-butylbenzene (1014-60-4), 2,4-di-*tert*-butylphenol (96-76-4), 2-ethylhexanoic acid (149-57-5), 2-mercaptobenzothiazole (149-30-4), benzoic acid (65–85-0), bis(2-ethylhexyl) phthalate (117-81-7), bis(4-chlorophenyl) sulfone (80-07-9), bisphenol A (80-05-7), butylhydroxymethylphenol (88-26-6), butylhydroxytoluene (128-37-0), caprolactam (105-60-2), dibenzylamine (103-49-1), drometrizole (2440-22-4), erucamide (112-84-5), Irganox 1010 (6683-19-8), Irganox 1076 (2082-79-3), Irganox 3114 (27676-62-6), oleamide (301-02-0), palmitic acid (57-10-3), stearic acid (57-11-4), and tris(2,4-di-*tert*-butylphenyl) phosphate (95906-11-9). Because the analytes were present in concentrations of 50 µg/mL, the mix was diluted to concentrations of 250 ng/mL and 750 ng/mL. The internal standards diphenylamine-D_10_ and stearic acid-D_35_ were added in concentrations of 200 ng/mL.

## Results and discussion

### Method characterization

To evaluate the performance, the LC-QTOF-MS/MS method was characterized for the 25 analytes and the 9 labelled standards, as displayed in Table 1. The choice of analytes was made due to their restriction by the MDR, leading to a toxicologically highly relevant selection. Their potential use in the production of polymers is listed in table S1 in the supplementary material. All analytes except four showed satisfying linearity in the tested concentration range from 50 to 500 ng/mL, with most having a linear response even beyond. Limit of detection (LOD) and limit of quantification (LOQ) were evaluated via signal-to-noise ratios (s/n) of 3 and 10, respectively.

**Table 1:** Figures of merit of the investigated compounds and the isotope-labelled standards

	LOD^A^	LLOQ^B^	ULOQ^C^	**Linearity R**^**2**^	RT	Inter-day precision (n = 3)^D^
	[ng/mL]				[min]	[RSD%]
1,3-diphenylurea-D_10_	50	100	500	0.9983	9.5	5.6
1-naphthol	1	5	10,000	0.9997	9.2	13
2,2′-methylenebis(4-methyl-6-tert-butylphenol)	1	5	1000	0.9978	16.7	4.7
2,4-tolylenediamine	1	5	500	0.9994	1.0	8.4
2,4,6-tri-*tert*-butylphenol	1	5	10,000	0.9990	17.4	10
2,6-dimethylphenol-D_3_	10	50	5000	0.9989	8.7	13
2-chlorophenol-D_4_	50	100	5000	0.9993	7.2	8.7
2-isopropylphenol	10	50	20,000	0.9994	10.1	10
2-naphthylamine	10	50	5000	0.9999	5.3	4.2
2-nitrophenol-D_4_	50	150	10,000	0.9994	3.8	13
2-phenylphenol	1	10	10,000	0.9997	10.4	9.8
2-*tert*-butylphenol	5	10	10,000	0.9997	11.6	3.2
3,3′-dimethoxybenzidine	50	100	500	0.9989	3.7	2.5
3,3′-dimethylbenzidine	5	50	100	0.9999	3.6	11
3-chlorophenol	5	10	20,000	0.9994	8.1	5.9
3-nitrophenol	1	5	20,000	0.9995	6.5	14
4,4′-methylenedianiline	50	100	5000	0.9957	1.4	11
4,4′-methylenebis(2-chloroaniline)	10	50	5000	0.9954	11.1	5.1
4,4′-methylenebis(2-methylaniline)	5	50	500	0.9999	3.8	12
4,4′-oxydianiline	10	50	500	0.9987	1.2	4.0
4-aminobiphenyl	5	50	500	0.9999	7.2	2.8
4-isopropylbenzoic acid	10	50	20,000	0.9995	4.9	12
4-*tert*-butylbenzoic acid	10	50	10,000	0.9997	5.5	8.7
Aniline Yellow	0.5	5	100	0.9997	10.8	6.8
Basic Violet 3	0.5	5	1000	0.9999	10.0	8.2
bisphenol A	10	50	5000	0.9998	9.1	11
butylhydroxytoluene-D_21_	1	10	10,000	0.9918	16.0	6.6
dicyclohexylphthalate-D_4_	10	50	1000	0.9956	16.2	13
diphenylamine-D_10_	50	100	1000	0.9992	11.9	5.7
Disperse Blue 1	10	100	1000	0.9999	5.0	4.0
Irgacure 907	1	5	100	0.9990	6.9	4.7
*m*-toluidine-D_3_	50	100	5000	0.9997	2.2	4.4
Michler’s ketone	0.5	5	100	0.9971	11.7	6.3
phenoxybenzoic acid-^13^C_6_	50	150	10,000	0.9982	5.5	7.3

^A^Limit of detection determined at s/n 3

^B^Lower limit of quantification determined at s/n 10

^C^Upper limit of quantification determined via evaluation of the regression curve

^D^Precision of peak areas determined from duplicate measurements at three concentration levels (100, 250, and 500 ng/mL) on three different days

**Fig. 1:**
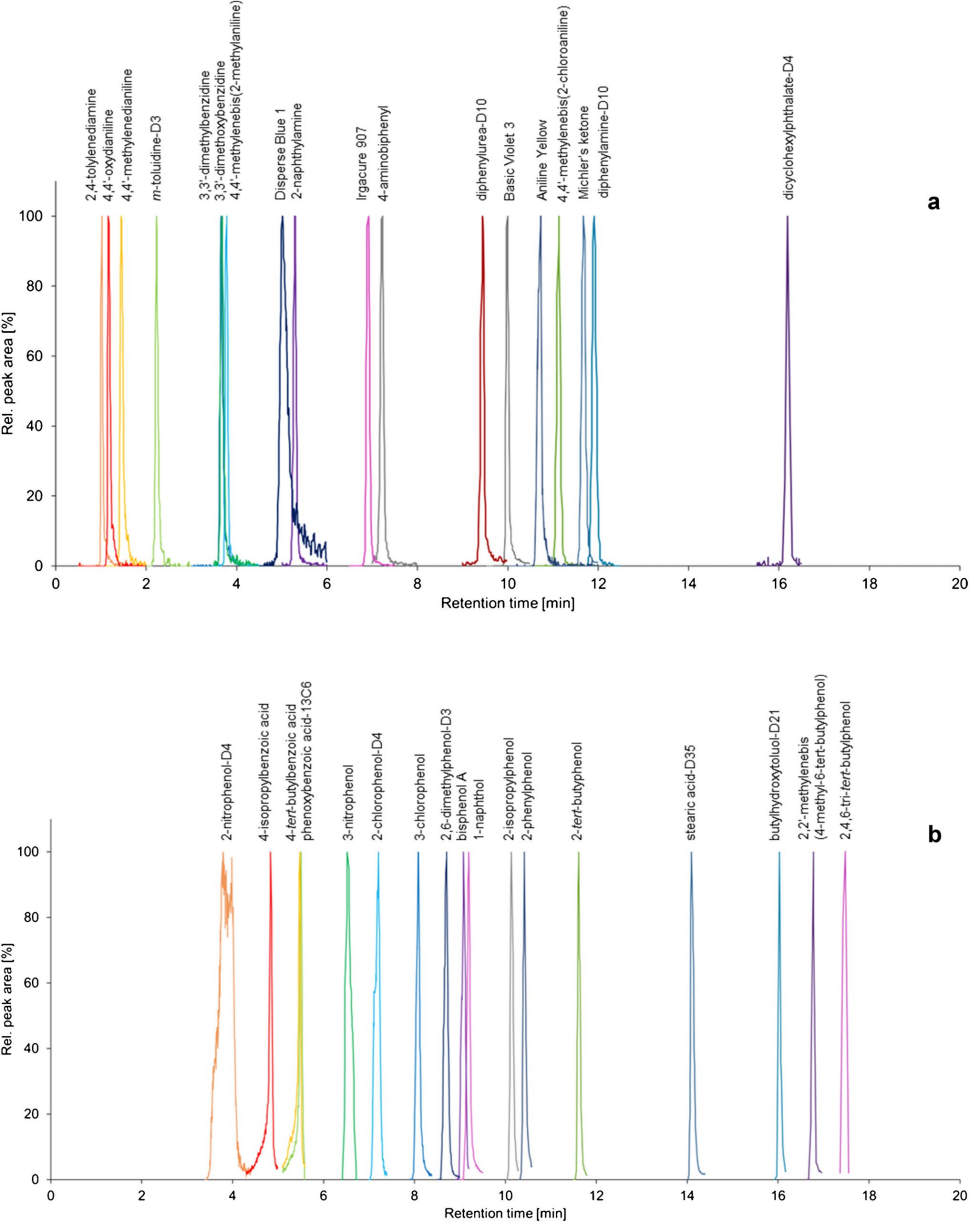
Extracted ion chromatograms displaying the normalized peaks of the analytes and the isotope-labelled surrogates. (**a**) Compounds measured in ESI+ mode. (**b**) Compounds measured in ESI- mode

Chromatograms showing the normalized peak areas of both the analytes and the isotope-labelled surrogates are displayed in Fig. 1. Co-elution of 3,3′-dimethoxybenzidine and 3,3′-dimethylbenzidine as well as other partial co-elutions were circumvented by preparing two analyte mixes and measuring them separately.

### Quantification with internal surrogates

#### Internal surrogate matched by retention time

Quantification without authentic reference standards is typically done as a one-point calibration via the internal surrogate (see formula S3). One way to match an analyte to a surrogate is to use the closest eluting surrogate. Ideally, they would have an equal or similar response factor. This assumption is mostly true for the analytes that are matched to *m*-toluidine-D_3_ as well as those matched to 1,3-diphenylurea-D_10_ for ESI+ mode. Those are mainly compounds that elute earlier and have a lower response factor.

The accuracy of the quantification is evaluated by a fold base quantification [19]. To better compare results, it is not differentiated between over- or underestimating the concentration:

1 $$\mathrm{if}\;{\mathrm c}_{\mathrm{predicted}}>{\mathrm c}_{\mathrm{spiked}}:\;\mathrm{quantification}\;\operatorname{error}=\frac{c_{predicted}}{c_{spiked}}$$

2 $$\mathrm{if}\;{\mathrm c}_{\mathrm{predicted}}<{\mathrm c}_{\mathrm{spiked}}:\;\mathrm{quantification}\;\operatorname{error}=\frac{c_{spiked}}{c_{predicted}}$$

A value of 1.0 times would therefore mean the lowest bias. Especially when testing against a threshold (as is the case for compliance with, e.g., MDR), a maximum quantification error of 2.0 times can be considered adequate, though it is common to find errors of up to 4.0 times or higher when using LC-MS [20, 27–29].

**Fig. 2:**
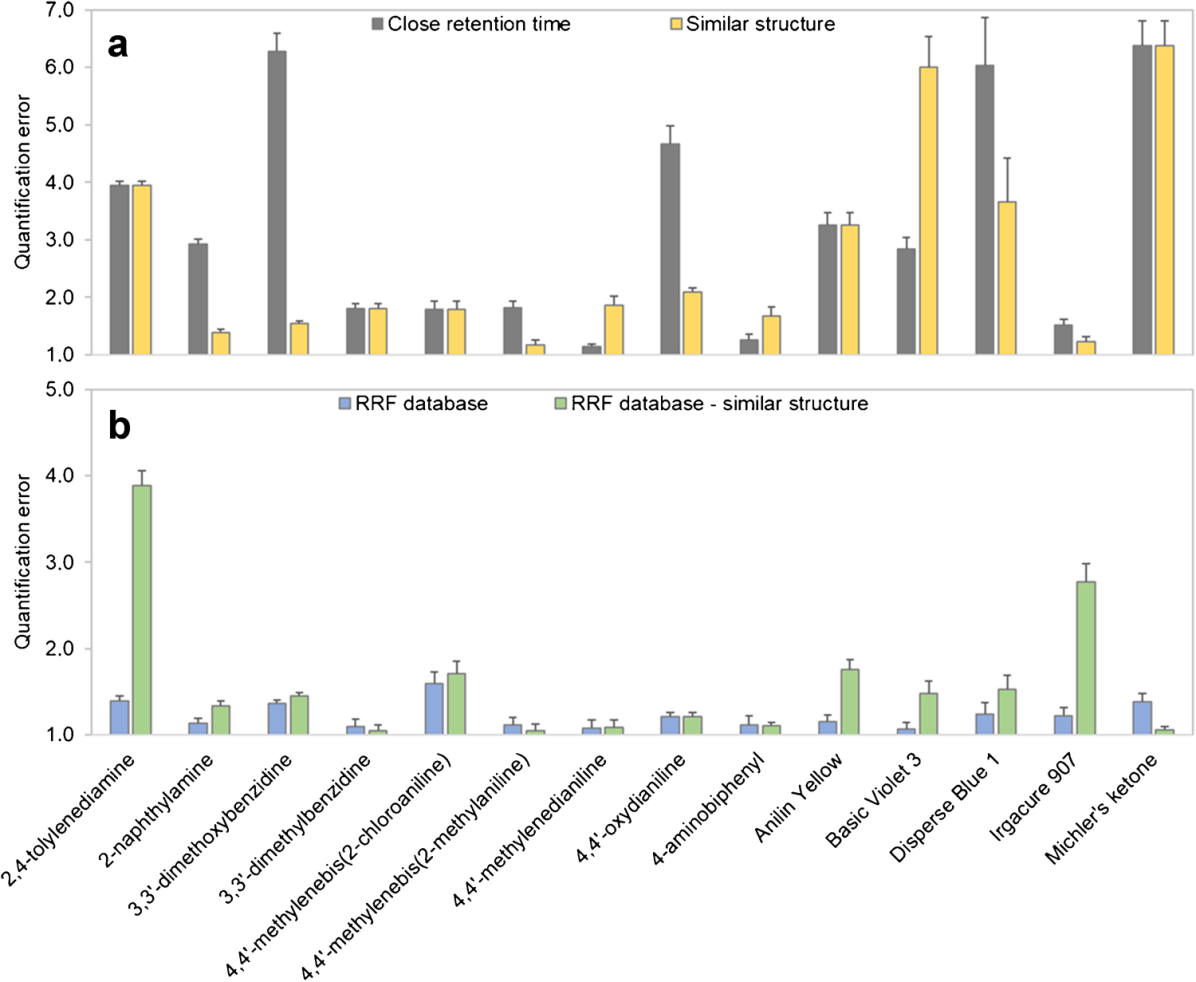
Comparison between quantification results in ESI+ mode. The bars represent the quantification errors as factors, with a value of 1 meaning the predicted concentration equals the spiked concentration. (**a**) Analytes were quantified using the closest eluting internal surrogate (gray) and a structurally similar internal surrogate (yellow). (**b**) Analytes were quantified using the respective analyte’s RRF (blue) and using the RRF of a structurally similar compound (green)

**Fig. 3:**
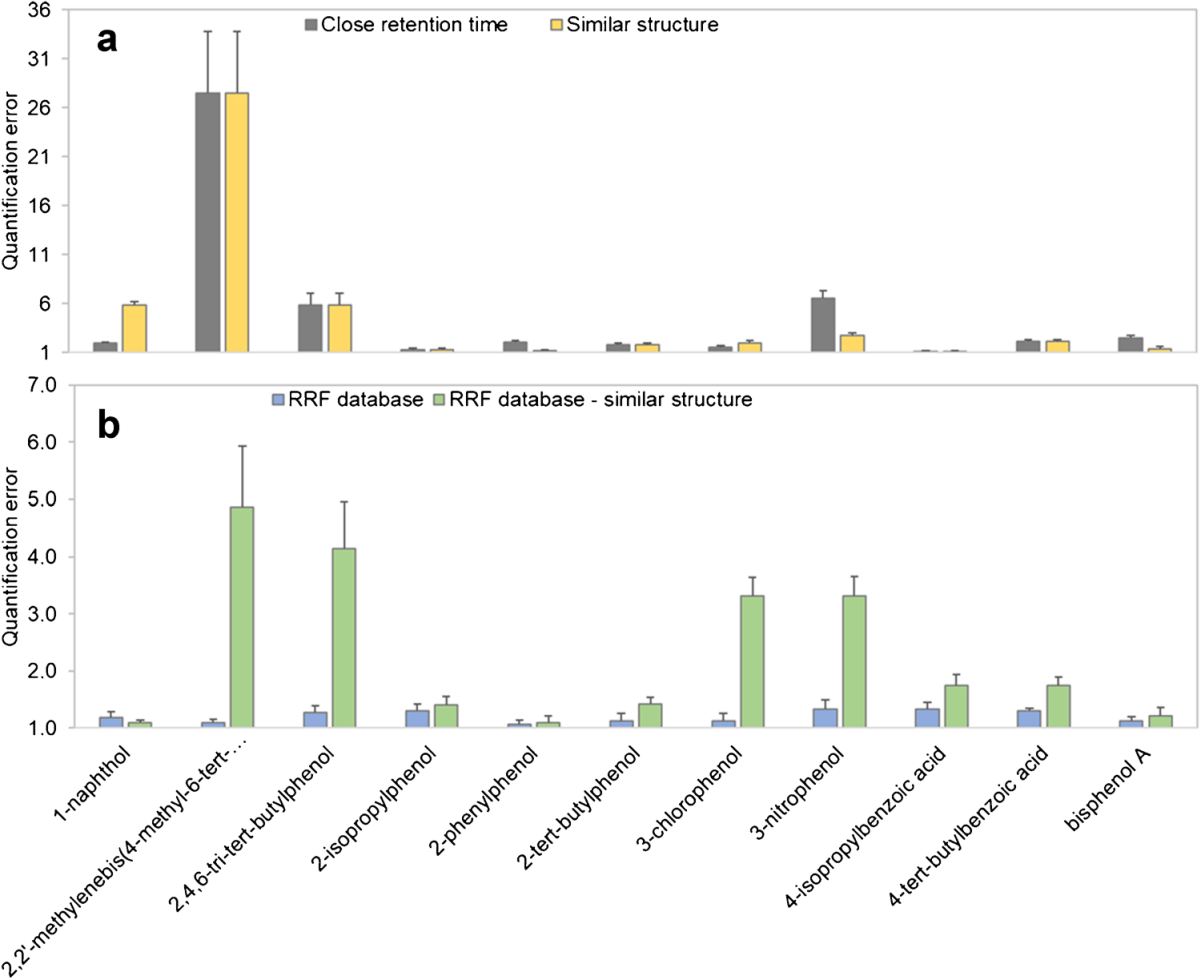
Comparison between quantification results in ESI- mode. The bars represent the quantification errors as factors, with a value of 1 meaning the predicted concentration equals the spiked concentration. (**a**) Analytes were quantified using the closest eluting internal surrogate (gray) and a structurally similar internal surrogate (yellow). (**b**) Analytes were quantified using the respective analyte’s RRF (blue) and using the RRF of a structurally similar compound (green)

Quantifying the analytes with the closest eluting surrogate leads to mixed results. Only 71% (ESI+) and 73% (ESI-) of the analytes can be quantified with a quantification error below 4.0 times, which is presented in Figs. 2a and 3a. A comparison of the performance of all quantification approaches can be found in Tables 2 and 3. For some analytes, such as 4,4′-methylenedianiline or 4-aminobiphenyl, the quantification error is as low as 1.1 and 1.3 times, respectively. The highest quantification errors of up to 28 times can be observed for 2,2′-methylenebis(4-methyl-6-*tert*-butylphenol). This analyte has by far the highest response factor and is therefore not well represented by any of the five internal surrogates used for ESI-.

**Table 2:** The summary of the performance of the four different quantification approaches with ESI+ mode (n = 14). The quantification errors are fold-based

Quantification approach	Internal surrogate matched by RT	Internal surrogate matched by structure	RRF of analyte from a database	RRF of structurally similar compound in database
Mean error	3.3 x	2.7 x	1.2 x	1.6 x
Median error	2.9 x	1.9 x	1.2 x	1.4 x
Maximum error	6.4 x	6.4 x	1.6 x	3.9 x
% of analytes with error < 2.0 x	43	57	100	86
% of analytes with error < 4.0 x	71	86	100	100

**Table 3:** The summary of the performance of the four different quantification approaches with ESI- mode (n = 11). The quantification errors are fold-based

Quantification approach	Internal surrogate matched by RT	Internal surrogate matched by structure	RRF of analyte from a database	RRF of structurally similar compound in database
Mean error	4.9 x	4.8 x	1.2 x	2.3 x
Median error	2.0 x	2.0 x	1.2 x	1.7 x
Maximum error	28 x	28 x	1.3 x	4.9 x
% of analytes with error < 2.0 x	45	45	100	64
% of analytes with error < 4.0 x	73	73	100	82

#### Internal surrogate matched by structure

Another possibility to match an analyte to a surrogate is to look for structural similarity. Similarity was evaluated by determining the atom pair (AP) Tanimoto similarity for each combination of analyte and surrogate using the ChemMine tool [25]. Tanimoto similarity is an easily available online tool and has already been used in literature. Matching the analyte to the structurally most similar surrogate, the response factors still deviate for a few compounds. This indicates that for this set of analytes, more surrogates with diverse structures would be needed.

When using a structurally similar surrogate for quantification, the mean quantification error improves slightly from 3.3 to 2.7 times for ESI+ mode and from 4.9 to 4.8 for ESI- mode, compared to using the closest eluting surrogate. This time, 86% (ESI+) of the analytes can be quantified with an error below 4.0 times, while for ESI- mode, the percentage stays the same.

For 11 of the analytes, the structurally most similar surrogate equals the one eluting the closest. The quantification errors are not significantly better (or worse), so this fact does not seem to have any effect.

These results further highlight that quantification accuracy depends highly on the chosen surrogates. However, laboratories are often restricted in their choice of suitable surrogates, especially if using isotope-labelled standards. They can be cost-intensive and the demands regarding the variety in retention time and response factors are not easy to fulfill. 

### Quantification with external surrogates in a RRF database

A possibility to use a wider range of surrogates is to measure them externally and thereby build up a RRF database. It circumvents the problem of co-elution with analytes, and the standards do not need to be isotope-labelled. If available, even the RRFs of the analytes themselves could be included in this database to allow a more accurate determination. For this, calibration levels of the analytes were prepared in the range from 10 to 500 ng/mL and measured on three different days, to obtain analyte-specific RRF values. As a reference point to the RFs, diphenylamine-D_10_ (ESI+) and stearic acid-D_35_ (ESI­) were added as internal standards in a concentration of 200 ng/mL to each calibration sample. While four analytes showed limited linearity at higher concentrations, the influence on the obtained mean RRF values is low. Since a direct quantification was not conducted, this was deemed acceptable.

With this approach, two cases can be distinguished: in case 1 the analytes have been measured before and their RRFs are included in the database, while in case 2 the analytes are not included and have to be matched to other standards, preferably by structure similar to quantifying with internal surrogates. Quantification can then be done by taking the specific RRF value into account, and only one internal standard needs to be added (see formula S4).

To build up the RRF database, the RRF values of the 25 analytes were obtained at five concentration levels measured in triplicates on three different days, with an average variation of 7.9% expressed as relative standard deviation. The exact values can be found in table S2 in the supplementary material. The highest variations are observed for 2-phenylphenol, 2-*tert*-butylphenol, 3-nitrophenol, 4,4′-methylenebis(2-methylaniline), and 4-isopropylbenzoic acid with relative standard deviations between 12 and 13%.

#### Using the RRF of the analyte from a database

When case 1 is applicable, the quantification with the previously determined RRFs leads to very low quantification errors. 100% of the analytes can be quantified with an error less than 2.0 times, and the mean errors for both ESI+ and ESI- mode are 1.2 times. The above-mentioned higher variation for the RRF values for 2-phenylphenol, 2-*tert*-butylphenol, 3-nitrophenol, 4,4′-methylenebis(2-methylaniline), and 4-isopropylbenzoic acid has seemingly no negative impact on the quantification accuracy, as can be seen in Figs. 2b and 3b.

#### Using the RRF of a structurally similar compound in a database

Case 2 is simulated by not using the analyte’s own RRF value but instead the RRF of the structurally most similar analyte (calculated by AP Tanimoto similarity). Here, the mean quantification errors of 1.6 times (ESI+) and 2.3 times (ESI-) are much lower than using the isotope-labelled internal surrogates. 86% (ESI+) and 64% (ESI-) of all analytes can be quantified with an error below 2.0 times. With errors between 3.9 times and 4.9 times, 2,2′-methylenebis(4-methyl-6-*tert*-butylphenol), 2,4,6-tri-*tert*-butylphenol, and 2,4-tolylenediamine stand out, though it should be easily possible to find a different matching surrogate in the future, when expanding the RRF database.

**Fig. 4:**
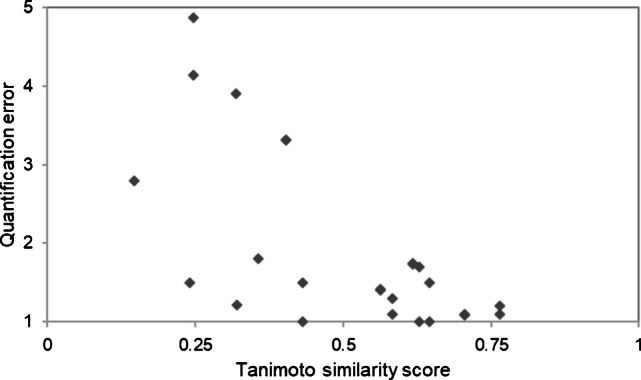
Diagram showing the relation between the AP Tanimoto similarity score and the quantification error. For a higher Tanimoto score, a trend for lower quantification errors can be observed

A trend can be observed that if the AP Tanimoto score is low, the quantification error is likely to be high (Fig. 4). A low score alone does not fully explain this, though it can be used as a hint to be more cautious when presenting the quantification results.

When using experimentally determined RRF values, it has to be kept in mind that they can change over time and should be remeasured at regular intervals [24, 30].

Tables 2 and 3 show the performance summary of the four tested quantification approaches. When quantifying with the analyte-specific RRFs, the best results can be achieved. This is only practicable if authentic reference standards are available, which cannot be assumed for the average extractable study. However, using the RRF of a structurally similar compound in the database yields satisfying results because of the possibility to use a greater variety of surrogates compared to using internal labelled surrogates.

### Influence of polymer matrices

The water extracts show little to no peaks in the chromatogram. Additionally, some of the analytes and the isotope-labelled standards are poorly soluble in water. That is why the data from the water extracts are not shown and are taken out of consideration. Apart from this, the polymer extracts show high variation in their chromatograms, even within a polymer type. Extracts with generally higher amounts of peaks in the chromatograms are the polyamide 6 (PA6), acrylonitrile butadiene styrene (ABS), ethylene propylene diene monomer (EPDM), and thermoplastic elastomer (TPE) extracts. Base peak chromatograms of the measured extracts can be found in the supplementary material (figures S1–S8). 

**Fig. 5:**
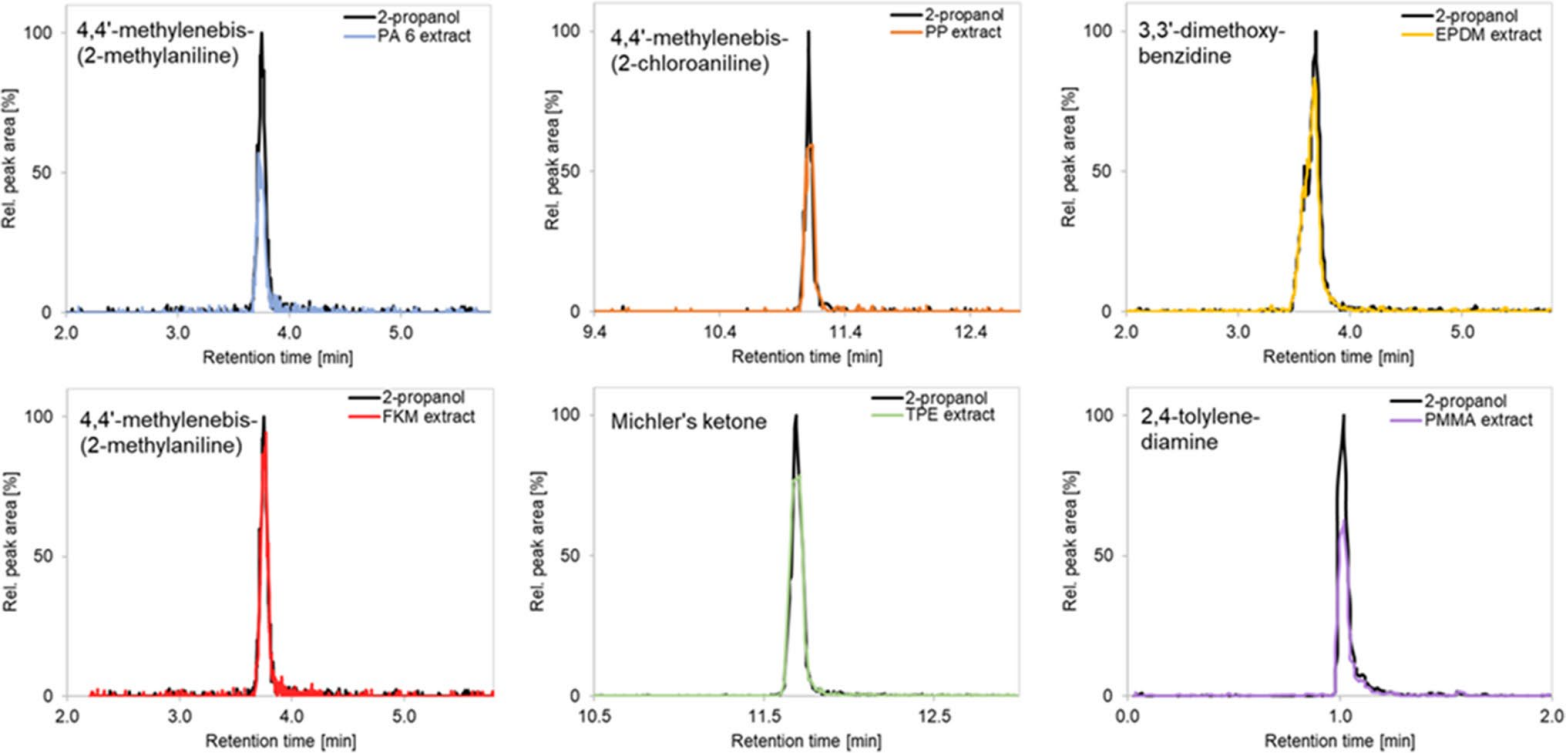
Sections of chromatograms comparing the peak areas of the analytes in pure 2-propanol (black) to those in polymer extracts (colored). Stronger ion suppression can be observed for 4,4′-methylenebis(2-methylaniline) in a PA6 extract (blue), 4,4′-methylenebis(2-chloroaniline) in a PP extract (orange), and 2,4-tolylenediamine in a PMMA extract (purple). In a FKM extract, however, the peak area of 4,4′-methylenebis(2-methylaniline) seems to not be significantly influenced by matrix components (red)

4,4′-Methylenebis(2-methylaniline) is a good example for an analyte suffering ion suppression, especially in a PA6 matrix. It co-elutes with an oligomer of caprolactam and when comparing the peak areas in pure solvent with those in matrix, it can be easily seen that suppression occurs (Fig. 5).

The quantification results for 4,4′-methylenebis(2-methylaniline) in the PA6 matrix are generally still acceptable, with errors ranging from 1.2 times (internal surrogate matched by RT) to 1.9 times (internal surrogate matched by structure and RRF of analyte). Quantifying with the closest eluting internal surrogate yields slightly better results. This partial elimination of matrix effects obviously only works if the response of the analyte and the surrogate matches well. Furthermore, the retention time of the surrogate has to be close to the retention time of the respective analyte since the effect of matrix components can change rapidly. This is why an elimination of matrix effects by a close-eluting internal surrogate cannot be assumed for most of the analytes.

As can be seen in the extracted ion chromatogram in Fig. 1, 2,4-tolylenediamine and 4,4′-oxydianiline elute close to the dead time of the method, where generally more signal suppression occurs. The quantification bias of these substances in polymeric matrices is not better or worse than for other compounds, so this fact does not seem to have an influence on the results.

**Fig. 6:**
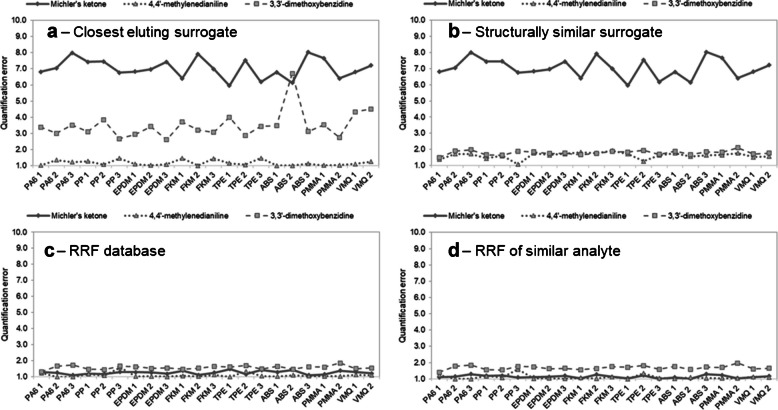
The diagrams show the quantification error with each quantification approach in dependence on the polymer matrix (in this case extracted by 2-propanol). Michler’s ketone, 3,3′-dimethoxybenzidine, and 4,4′-methylenedianiline were chosen to represent early and late eluting compounds, as well as compounds with higher and lower RFs. The quantification errors are mostly consistent across different matrices, especially for (**c**) and (**d**)

In general, the quantification errors are mostly consistent across different matrices, as can be seen in Fig. 6 for polymers extracted with 2-propanol (the toluene extracts show similar trends). While matrix effects can be observed and would influence a direct quantification to a certain extent, they seem to be neglectable when considering the error arising from the quantification. Especially the RRF database approaches show satisfying results, with none of the quantification errors worsening significantly. The low influence on the overall quantification performance was also observed by other authors for food and food contact matrices [13, 20, 31] as well as for matrix consisting of ground water [21].

### Quantification of a certified screening standard

Due to the lack of availability, no polymeric material could be analyzed as a certified reference material. Instead, a commercial screening standard containing analytes in defined concentrations was used for validating the concept. Since the quantification by the analyte’s own RRF yields a consistently satisfying bias, the focus laid on the quantification by the RRF of a structurally similar analyte.

While the screening standard contained 21 analytes, only 14 were detected in adequate intensity with the used methods. These analytes were then quantified with the RRFs of structurally similar surrogates (see Table 4). With a mean quantification error of 5.1 times, the bias is higher than for the 25 analytes with which this quantification strategy was developed. However, with a median of 1.5 times, it can be seen that the mean error is shifted towards a higher value mainly due to outliers. Especially erucamide and palmitic acid are quantified with high errors of 33 times and 11 times, respectively. The RRF database is mostly built up of aromatic compounds thus far. While the quantification concept works well for oleamide and stearic acid, two other aliphatic compounds, it is clear that the RRF database needs to be expanded with more structurally diverse analytes. Nevertheless, a good number of 64% of the analytes were quantified with an error below 2 times, confirming the decision to quantify with RRFs of structurally similar analytes in the future if no reference standards are available.

**Table 4:** The quantification errors obtained by quantifying reference material with the RRFs of structurally similar analytes

Analyte	AP Tanimoto score for external surrogate	Quantification error
2,4-di-*tert*-butylphenol	0.5000 (2,4,6-di-*tert*-butylphenol)	2.5
2-ethylhexanoic acid	0.0465 (2-isopropylphenol)	1.5
2-mercaptobenzothiazole	0.2346 (1-naphthol)	1.4
benzoic acid	0.4464 (3-nitrophenol)	6.4
bisphenol A	0.3210 (2-phenylphenol)	1.3
butylhydroxymethylphenol	0.2202 (1-naphthol)	1.4
butylhydroxytoluene	0.5730 (2,4,6-tri-*tert*-butylphenol)	1.4
caprolactam	0.0585 (Irgacure 907)	1.9
dibenzylamine	0.2651 (Aniline Yellow)	1.1
drometrizole	0.2663 (2-phenylphenol)	1.3
erucamide	0.0719 (Irgacure 907)	33
oleamide	0.0382 (4,4′-methylenebis(2-chloroaniline))	1.5
palmitic acid	0.0157 (2,2′-methylenebis(4-methyl-6-tert-butylphenol))	11
stearic acid	0.0145 (2,2′-methylenebis(4-methyl-6-tert-butylphenol))	5.6

## Conclusion

Conducting a quantification by using RRFs in a database allows to determine the concentration of all tested extractables with an error below 2.3 times. While best results are achieved when the analyte’s own RRF is available in the database, matching the analyte to a structurally similar compound in the database still provides mostly accurate results. A key limitation of the present study is the dependence on structural similarity for surrogate selection, which may introduce increased uncertainty for compounds that are poorly represented in the database. In addition, the current RRF database is biased towards aromatic compounds and requires further expansion to ensure broader applicability. In the future, it might be of interest to use more than one surrogate for quantification to reduce the impact of possible outliers. The RRF database approach will be expanded to include a broader range of structurally diverse extractables, making it possible to quantify even more extractables with satisfying accuracy. Since an unequivocal structure elucidation can be a huge challenge in non-target analysis, this is the major limitation of this strategy, and in prospective, additional parameters to match an analyte to a surrogate might need to be investigated to circumvent this challenge. Furthermore, it can be concluded that matrix effects do not significantly influence the quantification results, as the quantification errors remain mostly consistent across different polymer matrices.

## Supplementary Information

Below is the link to the electronic supplementary material.

Supplementary file1 (DOCX 514 KB)

## Data Availability

All relevant data generated or analyzed during this study are included in this published article and its supplementary information. Additional data may be available upon request.
