# Supplementary material for: Comparison of approaches for quantifying extractables in a variety of polymeric medical device materials with LC-MS/MS
Source: Anal Bioanal Chem. 2026 Mar 31;418(12):3665–77. doi: 10.1007/s00216-026-06471-9 (PMC13221373; doi:10.1007/s00216-026-06471-9)
Supplement: Supplementary file 1 — Supplementary file1 (DOCX 514 KB) [file 216_2026_6471_MOESM1_ESM.docx]

**Comparison of approaches for quantifying extractables in a variety of polymeric medical device materials with LC-MS/MS**

Anneke L. Niehuus ^1,2^, Max Renneisen ^1^, Sascha Reinschmiedt ^1^, Denise Sievers ^1^, Oliver J. Schmitz ^2*^, Sven W. Meckelmann ^2*^

_1_ Drägerwerk AG & Co. KGaA, Moislinger Allee 53-55, 23558 Lübeck, Germany

_2_ Applied Analytical Chemistry, University of Duisburg-Essen, Universitätsstrasse 5, 45141 Essen, Germany

*Corresponding author

# **Supplementary material**

**Formula S1.** Calculation of the response factor (RF).

${RF}_{analyte}=\frac{{peak area}_{analyte}}{c_{analyte}}$

**Formula S2.** Calculation of the relative response factor (RRF).

${RRF}_{analyte}=\frac{{RF}_{analyte}}{{RF}_{internal standard}}$

**Formula S3.** Determination of the concentration via the response of an internal surrogate.

$c_{analyte}=c_{surrogate}\times\frac{peak a{rea}_{analyte}}{peak {area}_{surrogate}}$

**Formula S4.** Determination of the concentration via the RRF in a database.

$c_{analyte}=\frac{{peak area}_{analyte}}{{RRF}_{analyte or surrogate}}\times\frac{c_{internal standard}}{{peak area}_{internal standard}}$

**Table S1.** Information on the structure of the analytes and the surrogates, their potential use in polymers, as well as the Tanimoto scores for the structurally most similar compound.

| compound | CAS-No. | structure | potential use in polymer production | AP Tanimoto score | |
| --- | --- | --- | --- | --- | --- |
|  |  |  |  | for internal surrogate | for external surrogate |
| 1,3-diphenylurea-D_10_ | 108009-46-7 | 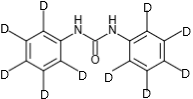 | n/a | n/a | n/a |
| 1-naphthol | 90-15-3 | 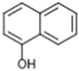 | intermediate | 0.3387  (2-chlorophenol-D_4_) | 0.7051  (2-phenylphenol) |
| 2,2’-methylenebis(4-methyl-6-tert-butylphenol) | 119-47-1 | 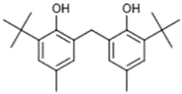 | antioxidant | 0.3291  (butylhydroxytoluene-D_21_) | 0.2460  (2,4,6-tri-*tert*-butylphenol) |
| 2,4-tolylenediamine | 95-80-7 | 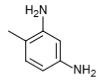 | intermediate | 0.4545  (*m*-toluidine-D_3_) | 0.3188  (2-naphthylamine) |
| 2,4,6-tri-*tert*-butylphenol | 732-26-3 | 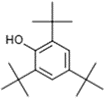 | intermediate for antioxidants | 0.5730  (butylhydroxytoluene-D_21_) | 0.2460  (2,2’-methylenebis(4-methyl-6-*tert*-butylphenol)) |
| 2,6-dimethylphenol-D_3_ | 576-26-1 (unlabeled) | 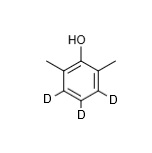 | n/a | n/a | n/a |
| 2-chlorophenol-D_4_ | 93951-73-6 | 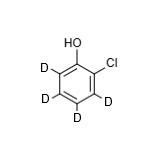 | n/a | n/a | n/a |
| 2-isopropylphenol | 88-69-7 | 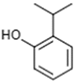 | contaminant in the production of bisphenol A | 0.4464  (2,6-dimethylphenol-D_3_) | 0.5625  (2-*tert*-butylphenol) |
| 2-naphthylamine | 91-59-8 | 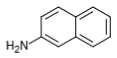 | antioxidant | 0.3434  (diphenylamine-D_10_) | 0.5833  (4-aminobiphenyl) |
| 2-nitrophenol-D_4_ | 93951-78-1 | 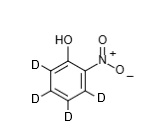 | n/a | n/a | n/a |
| 2-phenylphenol | 90-43-7 | 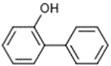 | intermediate for fire retardants | 0.4776  (phenoxybenzoic acid-^13^C_6_) | 0.7051  (1-naphthol) |
| 2-*tert*-butylphenol | 88-18-6 | 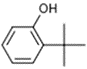 | intermediate for antioxidants | 0.3788  (2,6-dimethylphenol-D_3_) | 0.4464  (2-isopropylphenol) |
| 3,3’-dimethoxybenzidine | 119-90-4 | 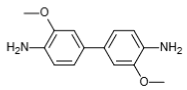 | intermediate for dyes | 0.1608  (diphenylamine-D_10_) | 0.6446  (3,3’-dimethylbenzidine) |
| 3,3’-dimethylbenzidine | 119-93-7 | 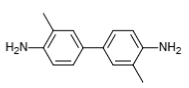 | intermediate for dyes | 0.1935  (*m*-toluidine-D_3_) | 0.6446  (3,3’-dimethoxybenzidine) |
| 3-chlorophenol | 108-43-0 | 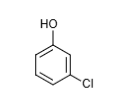 | intermediate for dyes | 0.5135  (2-chlorophenol-D_4_) | 0.4038  (3-nitrophenol) |
| 3-nitrophenol | 554-84-7 | 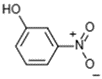 | intermediate | 0.5517  (2-nitrophenol-D_4_) | 0.4038  (3-chlorophenol) |
| 4,4’-methylenebis(2-chloroaniline) | 101-14-4 | 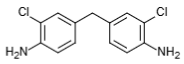 | curing agent | 0.1889  (diphenylamine-D_10_) | 0.6287  (4,4’-methylenebis(2-methylaniline)) |
| 4,4’-methylenebis(2-methylaniline) | 838-88-0 | 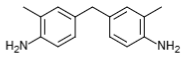 | curing agent | 0.1889  (diphenylamine-D_10_) | 0.6287  (4,4’-methylenebis(2-chloroaniline)) |
| 4,4’-methylenedianiline | 101-77-9 | 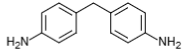 | monomer, curing agent | 0.3261  (diphenylamine-D_10_) | 0.7647  (4,4’-oxydianiline) |
| 4,4’-oxydianiline | 101-80-4 | 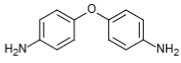 | intermediate | 0.3261  (diphenylamine-D_10_) | 0.7647  (4,4’-methylenedianiline) |
| 4-aminobiphenyl | 92-67-1 | 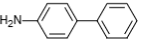 | antioxidant | 0.3929  (diphenylamine-D_10_) | 0.5833  (2-naphthylamine) |
| 4-isopropylbenzoic acid | 536-66-3 | 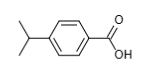 | intermediate | 0.2911  (2,6-dimethylphenol-D_3_) | 0.6180  (4-*tert*-butylbenzoic acid) |
| 4-*tert*-butylbenzoic acid | 98-73-7 | 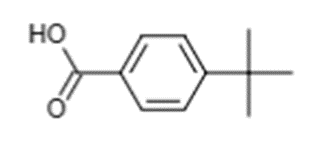 | intermediate | 0.2551  (2-nitrophenol-D_4_) | 0.6180  (4-isopropylbenzoic acid) |
| Aniline Yellow | 60-09-3 | 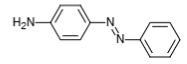 | dye | 0.3556  (diphenylamine-D_10_) | 0.3556  (4-aminobiphenyl) |
| Basic Violet 3 | 548-62-9 | 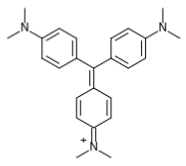 | dye | 0.1372  (diphenylamine-D_10_) | 0.4307  (Michler’s ketone) |
| bisphenol A | 80-05-7 | 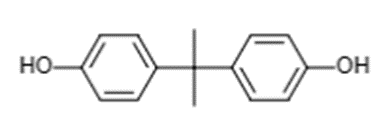 | monomer | 0.2073  (phenoxybenzoic acid-^13^C_6_) | 0.3210  (2-phenylphenol) |
| butylhydroxytoluene-D_21_ | 64502-99-4 | 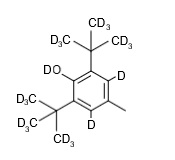 | n/a | n/a | n/a |
| dicyclohexylphthalate-D4 | 358731-25-6 | 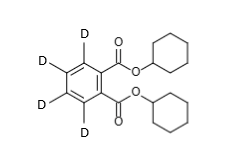 | n/a | n/a | n/a |
| diphenylamine-D_10_ | 37055-51-9 | 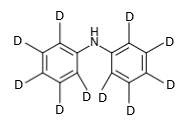 | n/a | n/a | n/a |
| Disperse Blue 1 | 2475-45-8 | 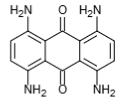 | dye | 0.1832  (1,3-diphenylurea-D_10_) | 0.2400  (3,3’-dimethylbenzidine) |
| Irgacure 907 | 71868-10-5 | 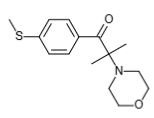 | photoinitiator | 0.1641  (dicyclohexylphthalate-D_4_) | 0.1460  (Michler’s ketone) |
| *m*-toluidine-D_3_ | 108-44-1  (unlabeled) | 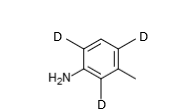 | n/a | n/a | n/a |
| Michler’s ketone | 90-94-8 | 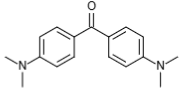 | intermediate for dyes | 0.2127  (diphenylamine-D_10_) | 0.4307  (Basic Violet 3) |
| phenoxybenzoic acid-^13^C_6_ | 1793055-05-6 | 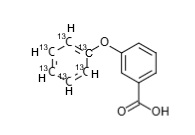 | n/a | n/a | n/a |

**Table S2.** The determined RRFs of the analytes. The RRFs were determined in relation to diphenylamine-D_10_ (ESI+) and stearic acid-D_35_ (ESI-).

| compound | mean RRF (n=3) |
| --- | --- |
| 1-naphthol | 1.97 ± 0.15 |
| 2,2’-methylenebis(6-*tert*-butyl-4-methylphenol) | 42.2 ± 2.4 |
| 2,4-tolylenediamine | 0.567 ± 0.048 |
| 2,4,6-tri-*tert*-butylphenol | 10.4 ± 1.1 |
| 2-isopropylphenol | 1.32 ± 0.11 |
| 2-naphthylamine | 1.58 ± 0.07 |
| 2-phenylphenol | 2.04 ± 0.24 |
| 2-*tert*-butylphenol | 1.85 ± 0.22 |
| 3,3’-dimethoxybenzidine | 0.879 ± 0.022 |
| 3,3’-dimethylbenzidine | 0.936 ± 0.099 |
| 3-chlorophenol | 0.67 ± 0.07 |
| 3-nitrophenol | 2.21 ± 0.27 |
| 4,4’-methylenebis(2-chloroaniline) | 0.893 ± 0.045 |
| 4,4’-methylenebis(2-methylaniline) | 0.936 ± 0.114 |
| 4,4’-methylenedianiline | 0.581 ± 0.062 |
| 4,4’-oxydianiline | 0.582 ± 0.023 |
| 4-aminobiphenyl | 1.85 ± 0.05 |
| 4-isopropylbenzoic acid | 0.97 ± 0.13 |
| 4-*tert*-butylbenzoic acid | 1.69 ± 0.14 |
| Aniline Yellow | 3.74 ± 0.25 |
| Basic Violet 3 | 6.21 ± 0.51 |
| bisphenol A | 2.25 ± 0.15 |
| Disperse Blue 1 | 0.761 ± 0.031 |
| Irgacure 907 | 3.89 ± 0.18 |
| Michler’s ketone | 8.82 ± 0.55 |


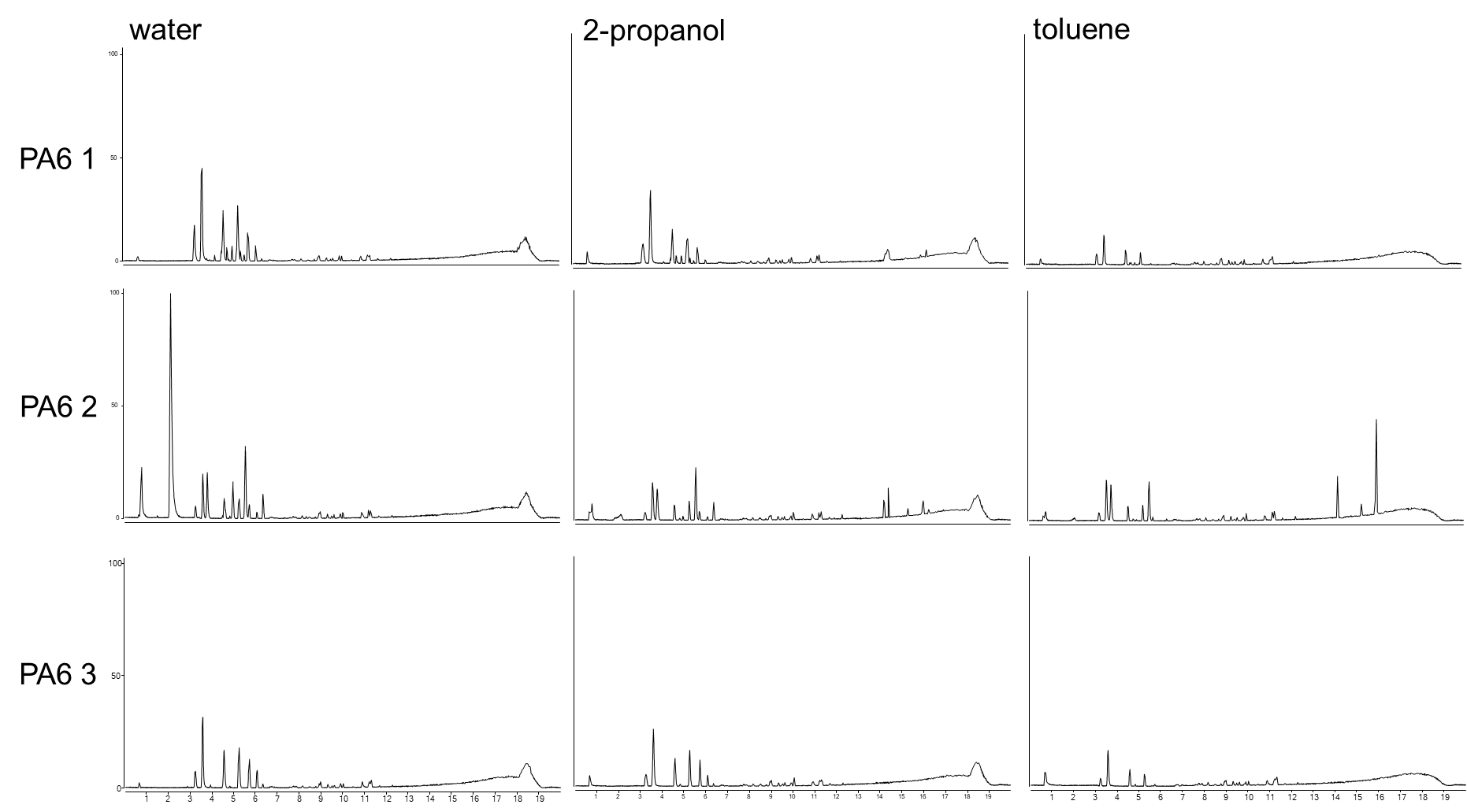


**Figure S1.** Base peak chromatograms of the extracts of three different polyamide 6 (PA6) materials.


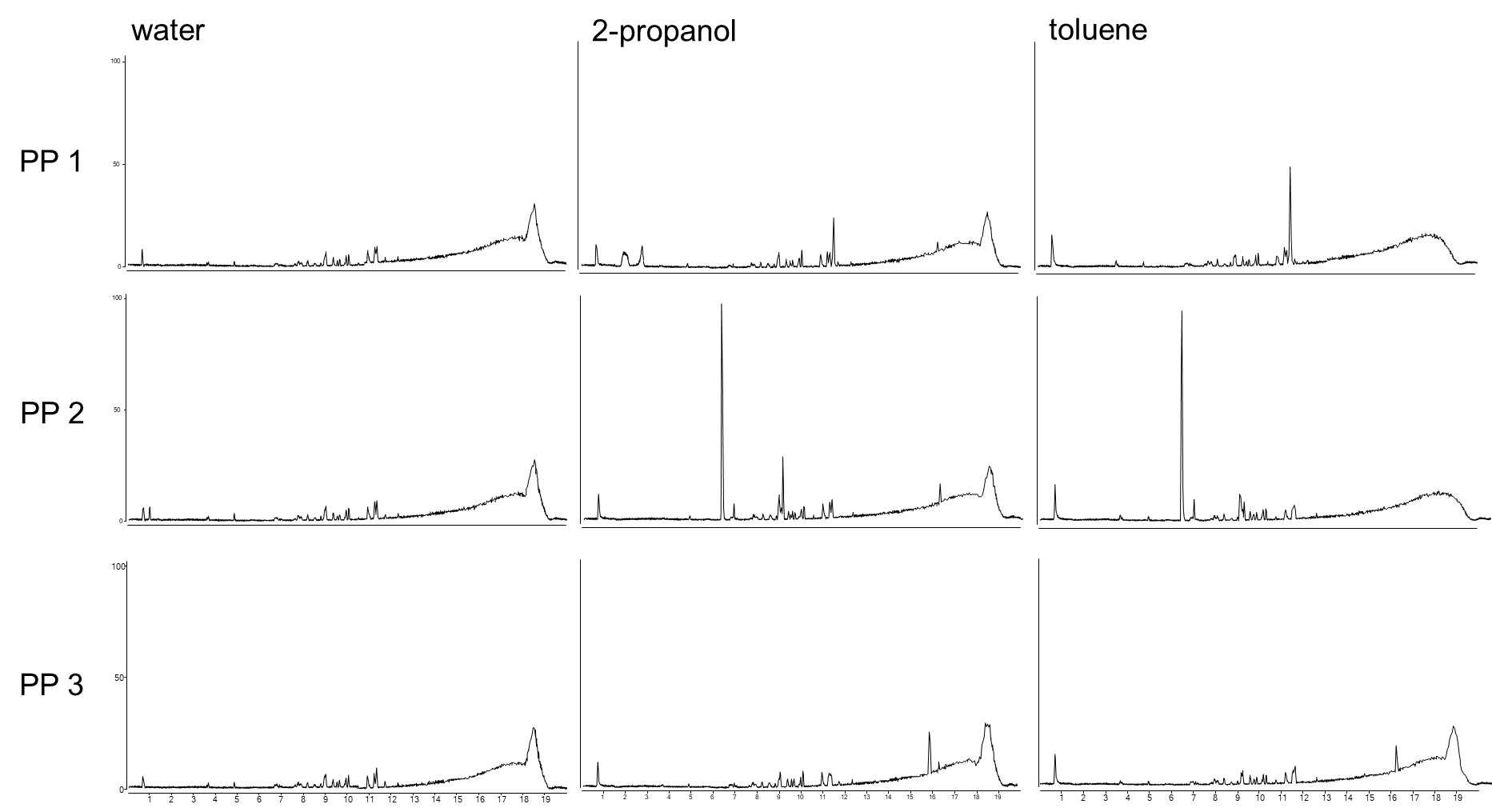


**Figure S2.** Base peak chromatograms of the extracts of three different polypropylene (PP) materials.


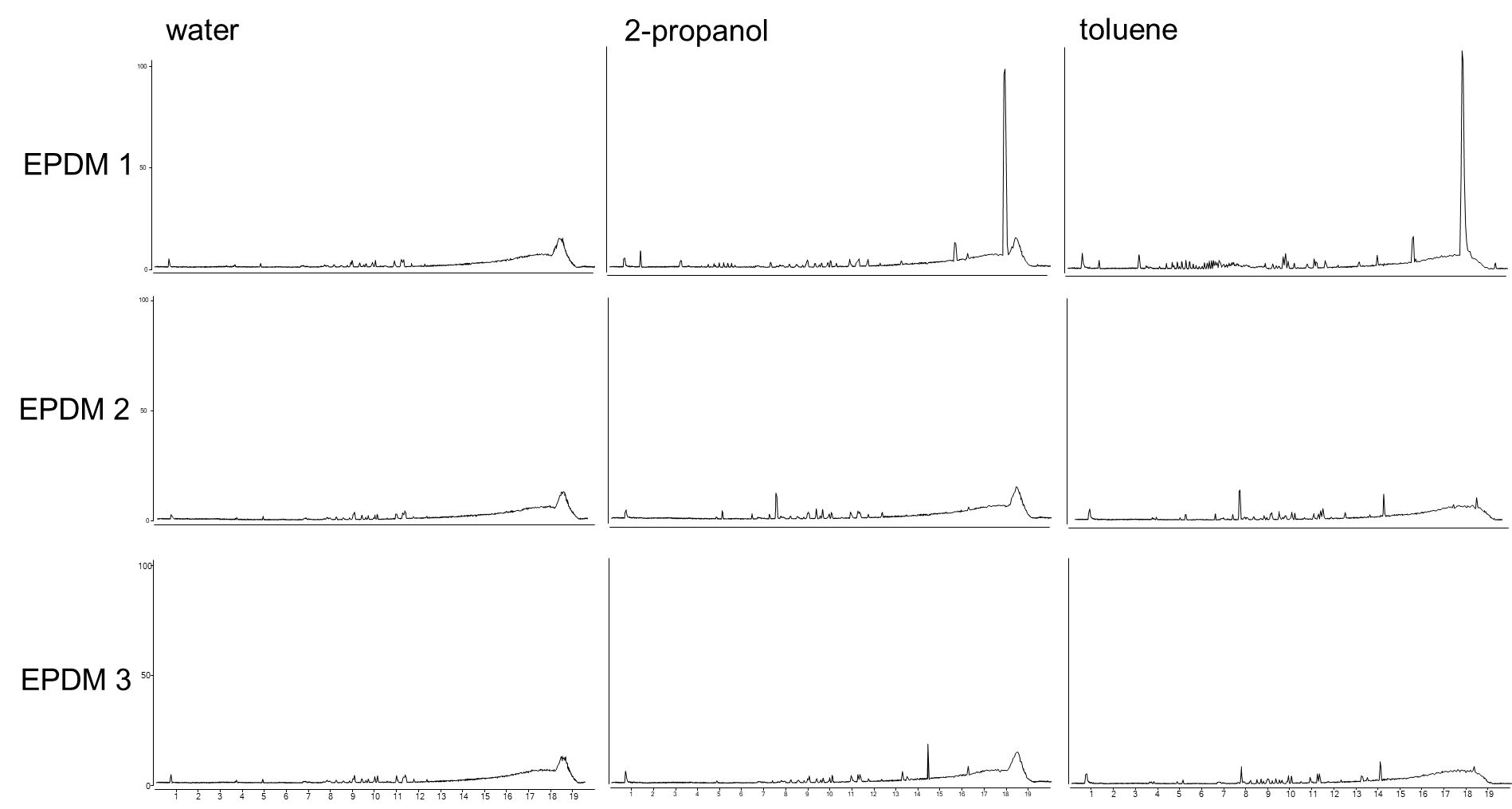


**Figure S3.** Base peak chromatograms of the extracts of three different ethylene propylene diene monomer rubber (EPDM) materials.


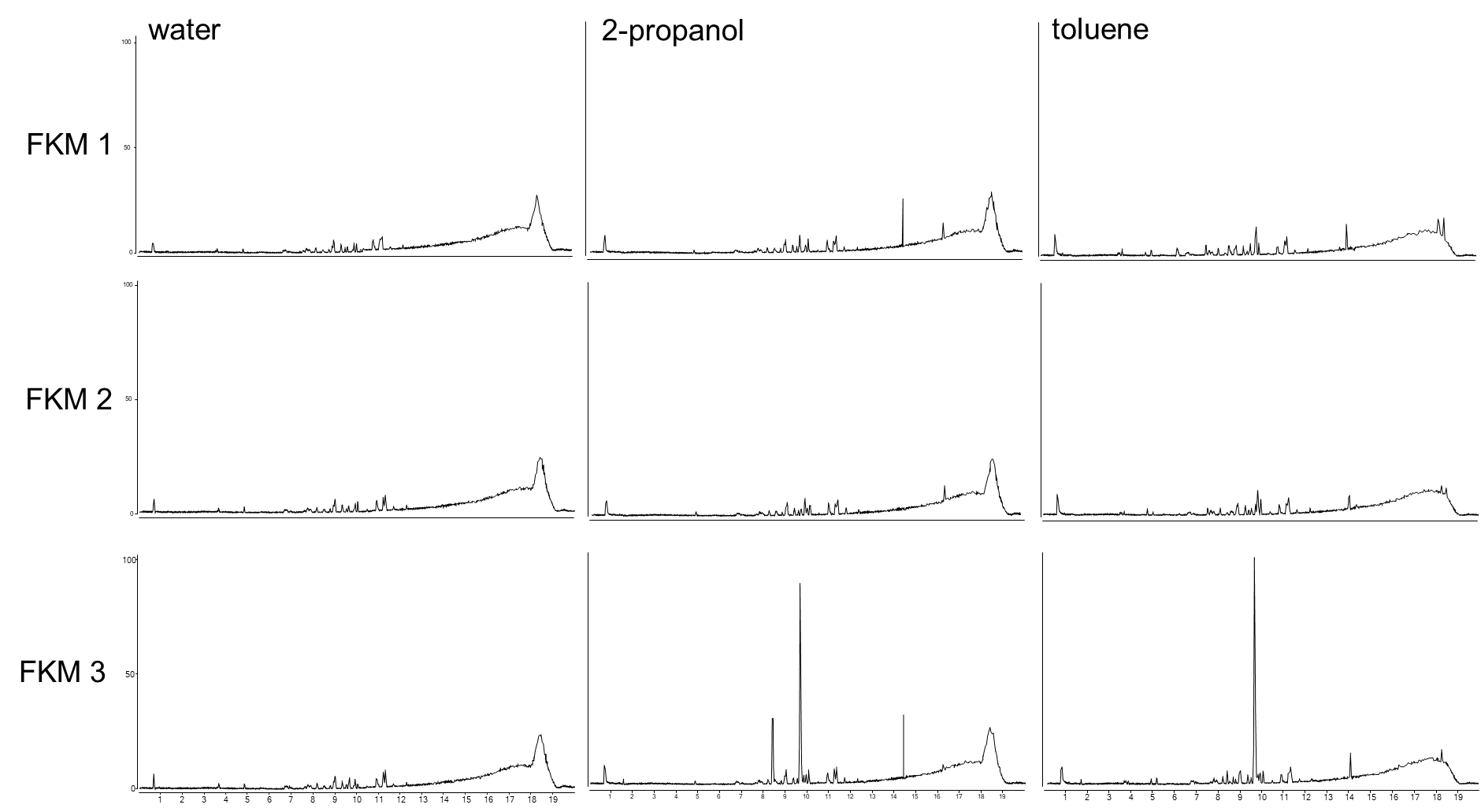


**Figure S4.** Base peak chromatograms of the extracts of three different fluoroelastomer (FKM) materials.


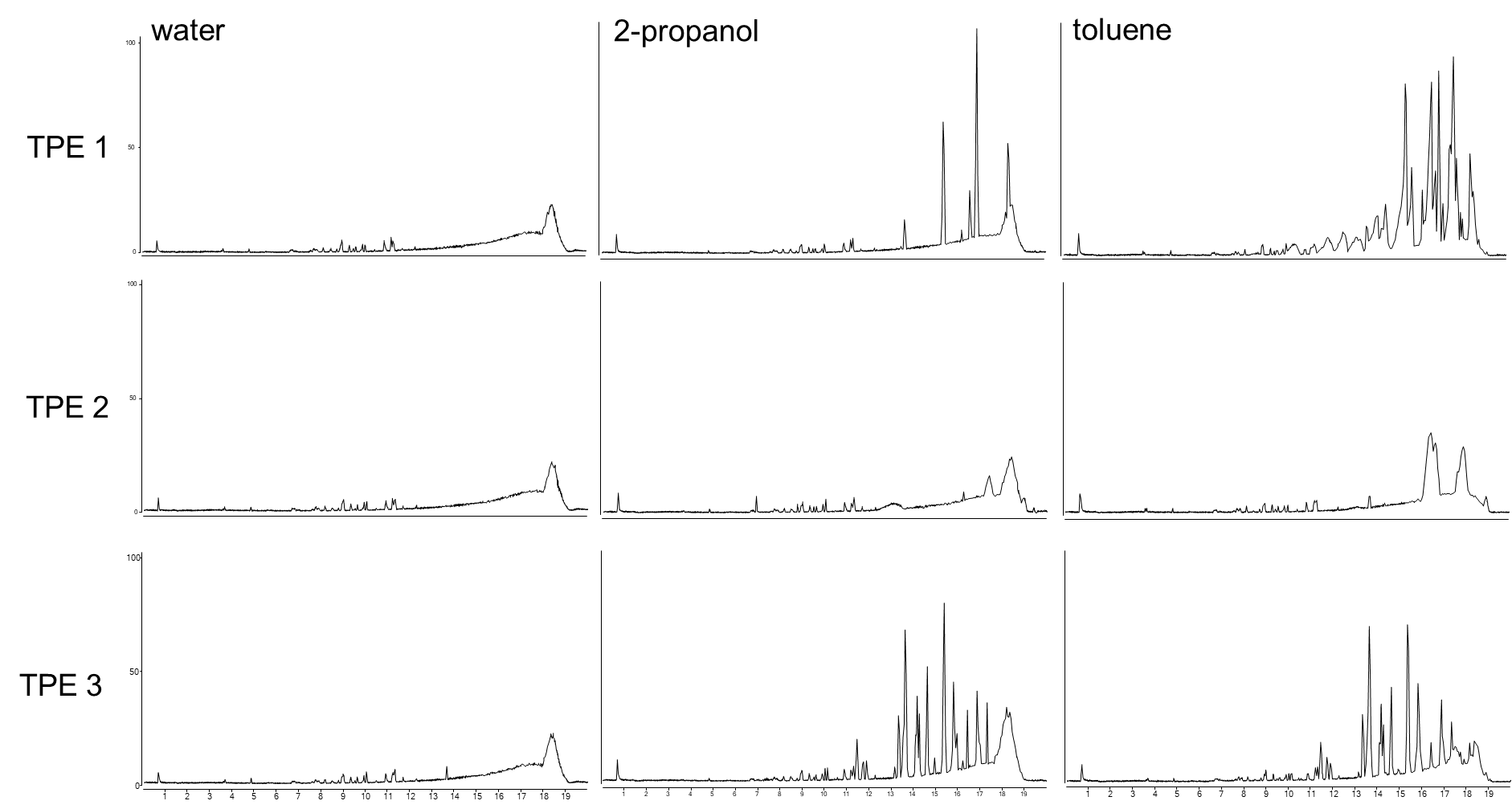


**Figure S5.** Base peak chromatograms of the extracts of three different thermoplastic elastomer (TPE) materials.


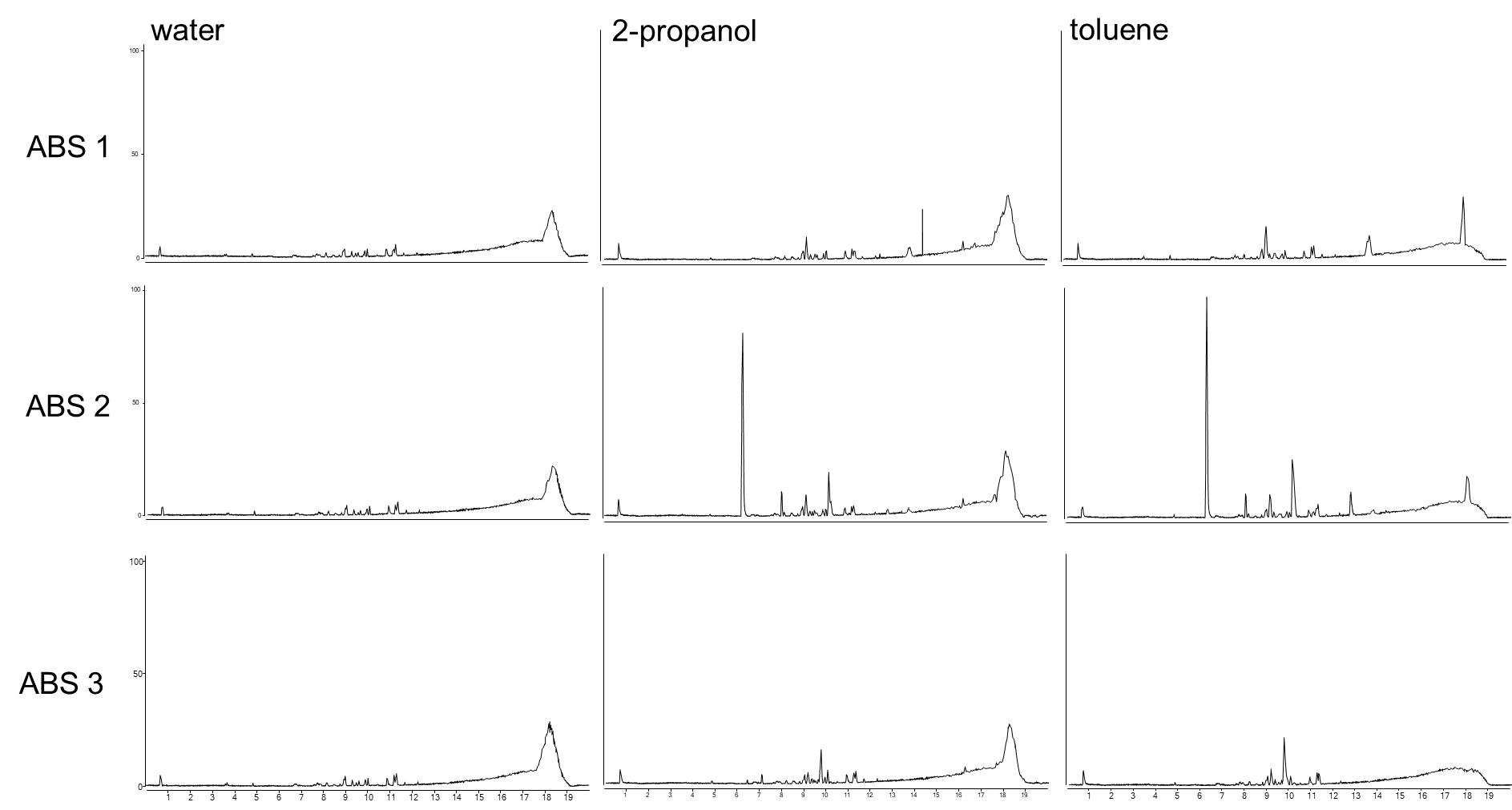


**Figure S6.** Base peak chromatograms of the extracts of three different acrylonitrile butadiene styrene (ABS) materials.


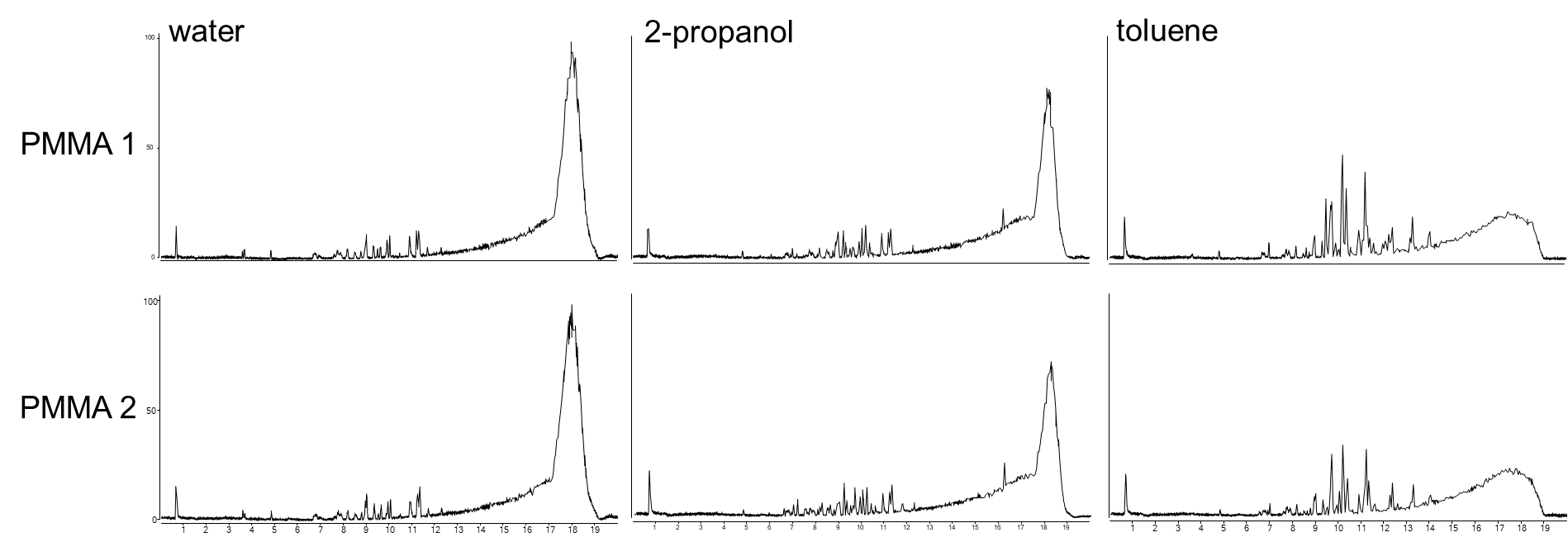


**Figure S7.** Base peak chromatograms of the extracts of two different polymethyl methacrylate (PMMA) materials.


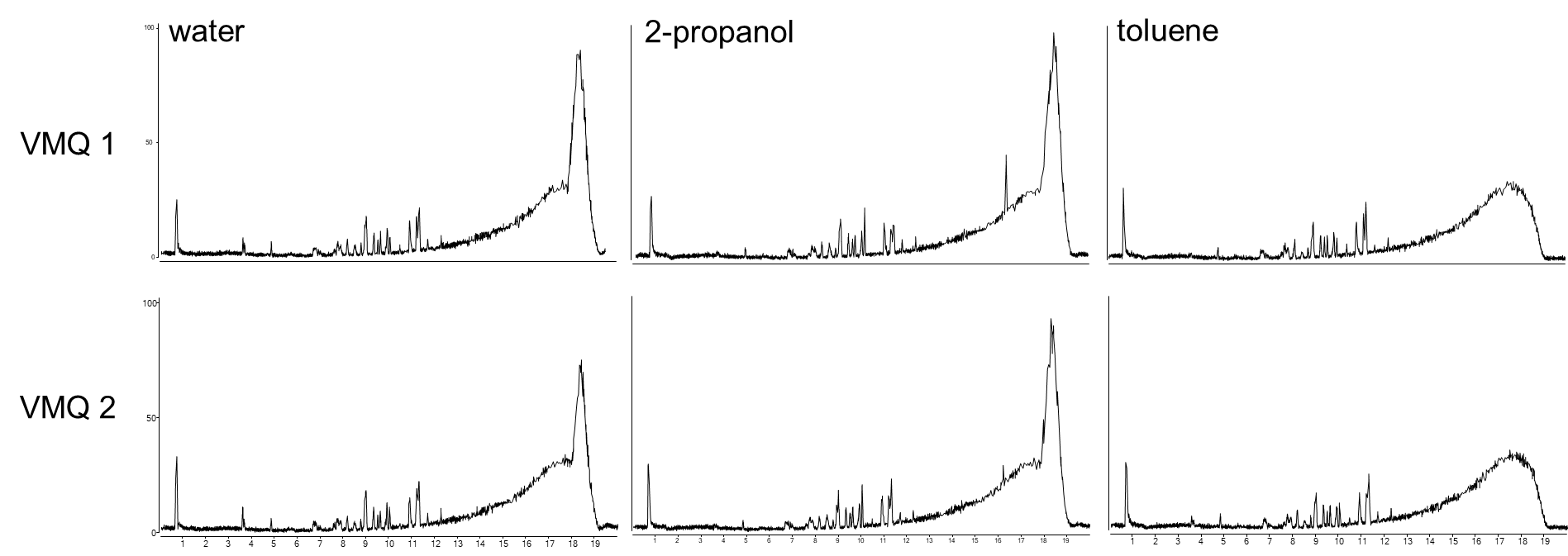


**Figure S8.** Base peak chromatograms of the extracts of two different silicone rubber (VMQ) materials.
